# Supplementary material for: Not a one-way road—Severity, progression and prevention of firework fears in dogs
Source: PLoS One. 2019 Sep 6;14(9):e0218150. doi: 10.1371/journal.pone.0218150 (PMC6730926; doi:10.1371/journal.pone.0218150)
Supplement: S1 File — (Table A) Relevant questions from the questionnaire survey. (Table B) Distribution of sex, neuter status and breed group (pure/ mixed)–where known—in dogs from different origins. (Table C) z-values for post-hoc tests of differences in Welfare Impaired scores in different breed groups. (Table D) p-values for post-hoc tests (adjusted for multiple testing) of differences in Welfare Impaired scores in different breed groups. (Table E) z-values for post-hoc tests comparing Welfare Impaired scores in dogs from different origins. (Table F) p-values for post-hoc tests (adjusted for multiple testing) comparing Welfare Impaired scores in dogs from different origins. (Table G) Results of a binomial model testing for the effects of health problems x age, source of dog, sex x neuter status, breed group, and age at acquisition on the occurrence of firework fears in dogs. Full model (AIC = 1229.63). (Table H) Results of a binomial model testing for the effects of health problems x age, source of dog, sex x neuter status and breed group on the occurrence of firework fears in dogs. Second model reduction (AIC = 1227.65). (Table I) Results of a binomial model testing for the effects of health problems x age, source of dog, sex, neuter status and breed group on the occurrence of firework fears in dogs. Third model reduction (AIC = 1225.95). (Table J) Results of a binomial model testing for the effects of health problems x age, neuter status and breed group on the occurrence of firework fears in dogs. Fifth model reduction (AIC = 1420.04 → higher than for the fourth model reduction presented in the manuscript as final reduced model). (DOCX) [file pone.0218150.s001.docx]

**S1 File. Supporting information**

**Table A.** Relevant questions from the questionnaire survey.

| **Questions** | **Response options** |
| --- | --- |
| Breed/ mix (if crossbreed, please indicate which breeds if known) | Open question |
| Date of birth according to the vaccination certificate/ pet passport | Date |
| Sex | Male - Female |
| Is your dog neutered? | Yes - No - Other |
| Source of your dog |  |
|  | From a small-scale breeder (< 5 litters a year) |
|  | From a large-scale breeder (5 or more litters a year) |
|  | From a private person whose dog had a litter |
|  | From a pet shop (later subsumed as 'other' as too few responses for this category) |
|  | Rehomed from a private person |
|  | Rehomed from a shelter in my country |
|  | Rehomed from a shelter abroad |
|  | Former street dog |
|  | Other |
| Age at adoption |  |
|  | < 3 months |
|  | 3-6 months |
|  | 7-11 months |
|  | 1-3 years |
|  | 4-6 years |
|  | 7-9 years |
|  | >9 years |
| Does your dog have any health problems? | Yes - No |
| Please rate your level of agreement with the following statements about your dog, ranging from “disagree strongly” to “agree strongly”. | Disagree strongly - tend to disagree - partly/ partly - tend to agree - agree strongly - I don't know |
|  | My dog is afraid of other dogs |
|  | My dog shows aggressive behaviour towards other dogs |
|  | My dog is afraid of people |
|  | My dog shows aggressive behaviour towards people |
|  | My dog shows separation related problems |
|  | My dog defends resources against other dogs |
|  | My dog defends resources against humans |
|  | My dog is hyperactive |
| Does your dog show fear in response to the following noises? | Disagree strongly - tend to disagree - partly/ partly - tend to agree - agree strongly - I don't know |
|  | Thunder |
|  | Gunshots |
|  | Motor noise |
|  | People shouting |
| Please rate your level of agreement with the following statement [The overall welfare of my dog is strongly compromised by fireworks] | Disagree strongly - tend to disagree - partly/ partly - tend to agree - agree strongly - I don't know |
| Have you administered medication or other calming products to your dog during the last firework event? | Yes - No |
| If yes, which ones? (Please select all applicable products). |  |
|  | Neutraceuticals (e.g. Zylkene, tryptophan etc.) |
|  | Pheromone products (Adaptil diffuser, spray or collar) |
|  | Herbal products |
|  | Homeopathic products |
|  | Bach flowers |
|  | Essential oils |
|  | Drugs (prescription medication available only from a veterinarian) |
| Please indicate the name of the product(s) used | Open question |
| Have you acquired help or advice about measures to prevent or alleviate your dog’s fear of fireworks? | Yes - No |
| If yes, from what source did you acquire help or advice? |  |
|  | From a vet |
|  | From a dog trainer or behaviourist/ behaviour specialist |
|  | On the internet |
|  | In magazines or books |
|  | From relatives, friends or acquaintances |
| Have you carried out any behavioural training specifically in order to treat or prevent noise fears in your dog? | Yes - No |
| If yes, when did you START with behavioural training against noise fears? |  |
|  | When my dog was a puppy and did not yet show a fear of fireworks |
|  | Before my dog showed fear of fireworks |
|  | When my dog showed first signs of fear of fireworks |
|  | When my dog showed pronounced signs of fear of fireworks |
| How long does it take until you dog’s behaviour is completely back to normal following a firework? |  |
|  | Immediately thereafter (or behaviour does not change due to fireworks) |
|  | Up to half an hour |
|  | Up to an hour |
|  | Up to three hours |
|  | By the next morning |
|  | In the course of the next day |
|  | Within three days |
| At what age did fear of fireworks first become apparent in your dog? |  |
|  | My dog is not afraid of fireworks |
|  | I adopted my dog as an adult when s/he already showed a fear of fireworks. |
|  | <1 year |
|  | 1 year |
|  | 2 years |
|  | 3 years |
|  | 4 years |
|  | 5 years |
|  | 6 years |
|  | 7 years |
|  | 8 years |
|  | 9 years |
|  | 10 years |
|  | 11 years |
|  | 12 years |
|  | 13 years |
|  | 14 years |
|  | 15 years |
| How has your dog’s fear of fireworks progressed in the last years? |  |
|  | My dog was never afraid of fireworks |
|  | The fear has improved greatly |
|  | The fear tends to have improved |
|  | The fear has remained the same |
|  | The fear tends to have become worse |
|  | The fear has become much worse |
|  | I don't know |

**Table B.** Distribution of sex, neuter status and breed group (pure/ mixed) – where known – in dogs from different origins.

|  | **Females** | | | **Males** | | | **Mixed breed vs single breed group  (both sexes)** | |
| --- | --- | --- | --- | --- | --- | --- | --- | --- |
| **Origin** | ***Total number of females*** | **Female intact (%)** | **Female neutered (%)** | ***Total number of males*** | **Male intact (%)** | **Male neutered (%)** | **Mixed breed (%)** | **Single breed group (%)** |
| **Big breeder** | *22* | 36 | 64 | *18* | 28 | 72 | 22 | 78 |
| **Homebred** | *10* | 80 | 20 | *2* | 100 | 0 | 20 | 80 |
| **Private breeder** | *69* | 28 | 72 | *98* | 37 | 63 | 45 | 55 |
| **Small breeder** | *150* | 43 | 57 | *199* | 46 | 54 | 10 | 90 |
| **Rehomed private** | *37* | 22 | 78 | *52* | 13 | 87 | 40 | 60 |
| **Rescue abroad** | *63* | 8 | 92 | *52* | 25 | 75 | 78 | 22 |
| **Rescue local** | *112* | 7 | 93 | *106* | 11 | 89 | 66 | 34 |
| **Other** | *15* | 20 | 80 | *12* | 25 | 75 | 37 | 63 |
| **Street dog** | *15* | 7 | 93 | *5* | 20 | 80 | 75 | 25 |

**Table C.** z-values for post-hoc tests of differences in Welfare Impaired scores in different breed groups.

|  | **companion** | **herding** | **spitz** | **molossian** | **terrier** | **pinscher** | **retriever** | **sighthound** | **flushing** | **other** | **hound** | **pointer** |
| --- | --- | --- | --- | --- | --- | --- | --- | --- | --- | --- | --- | --- |
| **mixed breed** | 3.53 | 1.62 | 1.16 | 4.50 | 1.18 | 1.66 | 5.01 | 0.75 | 2.66 | 1.97 | 3.62 | 0.47 |
| **companion** |  | 2.33 | 1.33 | 1.34 | 1.58 | 0.05 | 0.84 | 1.22 | 0.75 | 0.25 | 1.06 | 1.05 |
| **herding** |  |  | 0.33 | 3.49 | 0.23 | 1.17 | 3.59 | 0.12 | 2.08 | 1.44 | 2.83 | 0.00 |
| **spitz** |  |  |  | 2.38 | 0.11 | 0.87 | 2.08 | 0.12 | 1.61 | 1.09 | 2.05 | 0.18 |
| **molossian** |  |  |  |  | 2.67 | 0.76 | 0.66 | 2.14 | 0.18 | 0.61 | 0.10 | 1.81 |
| **terrier** |  |  |  |  |  | 0.97 | 2.43 | 0.04 | 1.76 | 1.20 | 2.27 | 0.11 |
| **pinscher** |  |  |  |  |  |  | 0.40 | 0.89 | 0.51 | 0.14 | 0.65 | 0.85 |
| **retriever** |  |  |  |  |  |  |  | 1.83 | 0.26 | 0.22 | 0.46 | 1.52 |
| **sighthound** |  |  |  |  |  |  |  |  | 1.55 | 1.09 | 1.89 | 0.08 |
| **flushing** |  |  |  |  |  |  |  |  |  | 0.37 | 0.10 | 1.41 |
| **other** |  |  |  |  |  |  |  |  |  |  | 0.50 | 1.02 |
| **hound** |  |  |  |  |  |  |  |  |  |  |  | 1.65 |

**Table D.** p-values for post-hoc tests (adjusted for multiple testing) of differences in Welfare Impaired scores in different breed groups.

|  | **companion** | **herding** | **spitz** | **molossian** | **terrier** | **pinscher** | **retriever** | **sighthound** | **flushing** | **other** | **hound** | **pointer** |
| --- | --- | --- | --- | --- | --- | --- | --- | --- | --- | --- | --- | --- |
| **mixed breed** | 0.03 | 1 | 1 | 0.0005 | 1 | 1 | 0.00004 | 1 | 0.60 | 1 | 0.02 | 1 |
| **companion** |  | 1 | 1 | 1 | 1 | 1 | 1 | 1 | 1 | 1 | 1 | 1 |
| **herding** |  |  | 1 | 0.04 | 1 | 1 | 0.03 | 1 | 1 | 1 | 0.37 | 1 |
| **spitz** |  |  |  | 1 | 1 | 1 | 1 | 1 | 1 | 1 | 1 | 1 |
| **molossian** |  |  |  |  | 0.56 | 1 | 1 | 1 | 1 | 1 | 1 | 1 |
| **terrier** |  |  |  |  |  | 1 | 1 | 1 | 1 | 1 | 1 | 1 |
| **pinscher** |  |  |  |  |  |  | 1 | 1 | 1 | 1 | 1 | 1 |
| **retriever** |  |  |  |  |  |  |  | 1 | 1 | 1 | 1 | 1 |
| **sighthound** |  |  |  |  |  |  |  |  | 1 | 1 | 1 | 1 |
| **flushing** |  |  |  |  |  |  |  |  |  | 1 | 1 | 1 |
| **other** |  |  |  |  |  |  |  |  |  |  | 1 | 1 |
| **hound** |  |  |  |  |  |  |  |  |  |  |  | 1 |

**Table E.** z-values for post-hoc tests comparing Welfare Impaired scores in dogs from different origins.

|  | **homebred** | **other** | **private breeder** | **rehomed private** | **rescue abroad** | **rescue local** | **small breeder** | **street dog** |
| --- | --- | --- | --- | --- | --- | --- | --- | --- |
| **big breeder** | 1.40 | 0.63 | 0.84 | 0.57 | 2.49 | 1.77 | 0.03 | 0.07 |
| **homebred** |  | 1.78 | 2.04 | 1.85 | 3.02 | 2.58 | 1.59 | 1.21 |
| **other** |  |  | 0.04 | 0.22 | 1.40 | 0.73 | 0.76 | 0.60 |
| **private breeder** |  |  |  | 0.30 | 2.55 | 1.53 | 1.52 | 0.71 |
| **rehomed private** |  |  |  |  | 2.46 | 1.56 | 0.87 | 0.52 |
| **rescue abroad** |  |  |  |  |  | 1.31 | 4.21 | 1.96 |
| **rescue local** |  |  |  |  |  |  | 3.48 | 1.39 |
| **small breeder** |  |  |  |  |  |  |  | 0.11 |

**Table F.** p-values for post-hoc tests (adjusted for multiple testing) comparing Welfare Impaired scores in dogs from different origins.

|  | **homebred** | **other** | **private breeder** | **rehomed private** | **rescue abroad** | **rescue local** | **small breeder** | **street dog** |
| --- | --- | --- | --- | --- | --- | --- | --- | --- |
| **big breeder** | 1 | 1 | 1 | 1 | 0.47 | 1 | 1 | 1 |
| **homebred** |  | 1 | 1 | 1 | 0.09 | 0.35 | 1 | 1 |
| **other** |  |  | 1 | 1 | 1 | 1 | 1 | 1 |
| **private breeder** |  |  |  | 1 | 0.39 | 1 | 1 | 1 |
| **rehomed private** |  |  |  |  | 0.49 | 1 | 1 | 1 |
| **rescue abroad** |  |  |  |  |  | 1 | 0.00 | 1 |
| **rescue local** |  |  |  |  |  |  | 0.02 | 1 |
| **small breeder** |  |  |  |  |  |  |  | 1 |

**Table G.** Results of a binomial model testing for the effects of health problems x age, source of dog, sex x neuter status, breed group, and age at acquisition on the occurrence of firework fears in dogs. First model reduction (AIC = 1229.63).

| **Predictor** | **Chi^2^ Likelihood ratio** | **Degrees of freedom** | **p** |
| --- | --- | --- | --- |
| **Health problems** | 16.18 | 1 | 0.00006 |
| **Age** | 35.20 | 10 | 0.000000003 |
| **Source of dog** | 12.73 | 1 | 0.24 |
| **Sex** | 1.01 | 1 | 0.32 |
| **Neuter status** | 0.30 | 1 | 0.58 |
| **Breed group** | 36.42 | 12 | 0.0003 |
| **Age acquired** | 0.01 | 1 | 0.91 |
| **Health problems x Age** | 15.67 | 1 | 0.00008 |
| **Sex x Neuter status** | 0.30 | 1 | 0.58 |

**Table H.** Results of a binomial model testing for the effects of health problems x age, source of dog, sex x neuter status and breed group on the occurrence of firework fears in dogs. Second model reduction (AIC = 1227.65).

| **Predictor** | **Chi^2^ Likelihood ratio** | **Degrees of freedom** | **p** |
| --- | --- | --- | --- |
| **Health problems** | 16.17 | 1 | 0.00005783 |
| **Age** | 35.43 | 10 | <0.00000001 |
| **Source of dog** | 12.9 | 1 | 0.23 |
| **Sex** | 1.00 | 1 | 0.31 |
| **Neuter status** | 0.40 | 1 | 0.53 |
| **Breed group** | 36.43 | 12 | 0.00028 |
| **Health problems x Age** | 15.66 | 1 | 0.00008 |
| **Sex x Neuter status** | 0.3 | 1 | 0.58 |

**Table I.** Results of a binomial model testing for the effects of health problems x age, source of dog, sex, neuter status and breed group on the occurrence of firework fears in dogs. Third model reduction (AIC = 1225.95).

| **Predictor** | **Chi^2^ Likelihood ratio** | **Degrees of freedom** | **p** |
| --- | --- | --- | --- |
| **Health problems** | 16.15 | 1 | 0.00006 |
| **Age** | 35.27 | 10 | <0.00000001 |
| **Source of dog** | 12.66 | 1 | 0.243 |
| **Sex** | 1.07 | 1 | 0.3 |
| **Neuter status** | 2.30 | 1 | 0.129 |
| **Breed group** | 36.56 | 12 | 0.0003 |
| **Health problems x Age** | 15.66 | 1 | 0.00008 |

**Table J.** Results of a binomial model testing for the effects of health problems x age, neuter status and breed group on the occurrence of firework fears in dogs. Fifth model reduction (AIC = 1420.04
🡪 higher than for the fourth model reduction presented in the manuscript as final reduced model).

| **Predictor** | **Chi^2^ Likelihood ratio** | **Degrees of freedom** | **p** |
| --- | --- | --- | --- |
| **Health problems** | 14.76 | 1 | 0.0001 |
| **Age** | 52.35 | 10 | <0.0000000000001 |
| **Neuter status** | 0.92 | 1 | 0.33 |
| **Breed group** | 57.29 | 12 | 0.00000007 |
| **Health problems x Age** | 15.70 | 1 | 0.00007 |
