# Supplementary figures and images for: Not a one-way road—Severity, progression and prevention of firework fears in dogs
Source: PLoS One. 2019 Sep 6;14(9):e0218150. doi: 10.1371/journal.pone.0218150 (PMC6730926; doi:10.1371/journal.pone.0218150)

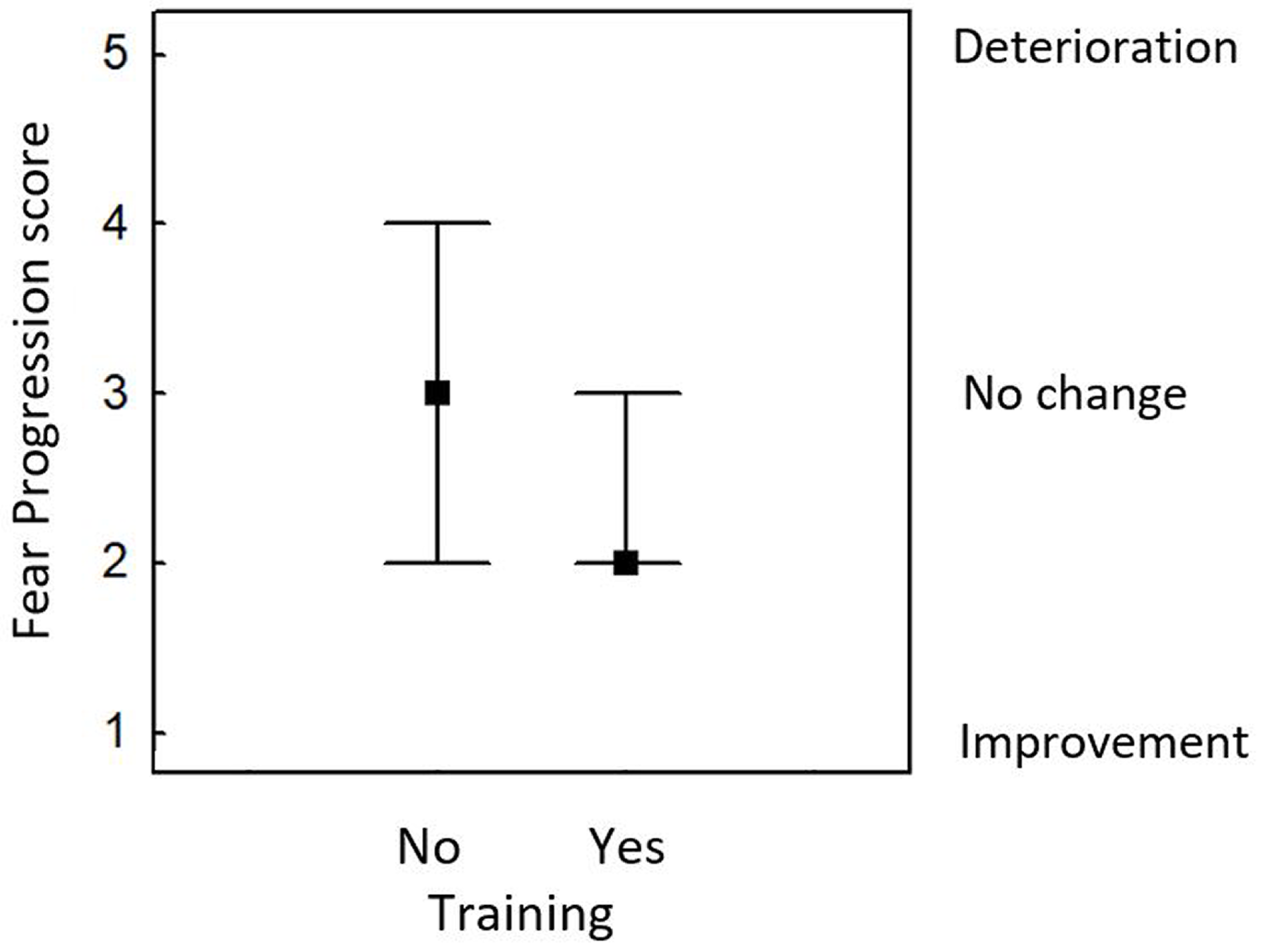

Supplement: S1 Fig — (TIF) [file pone.0218150.s002.tif]
